# Unveiling the complex landscape of successful weight loss: Perceived consequences and spontaneous self-concept insights

**DOI:** 10.1177/13591053251348301

**Published:** 2025-07-16

**Authors:** Filipa Pimenta, Inês Queiroz-Garcia, Cátia Damião, Raquel Rosas, Isabel Leal

**Affiliations:** 1 Ispa – Instituto Universitário/William James Center for Research, Portugal; 2 Ispa – Instituto Universitário, Portugal; 3 Davis Pier Consulting, Canada

**Keywords:** directed qualitative content analysis, negative consequences, positive consequences, self-concept, successful weight loss

## Abstract

This study explores the perceived positive and negative consequences, as well as the spontaneous self-concept of 60 individuals who have achieved successful weight loss. Using a descriptive-transversal, exploratory design and Directed Qualitative Content Analysis, semi-structured interviews were analyzed. A quantitative exploration of the associations between the emergent categories was conducted using multiple correspondence analysis, which revealed a four-factor model: (1) all gains (health, psychological and social) but the body image, (2) role-model and bodily efficacious with fallouts (psychological and family), (3) body satisfaction and sexual disinhibition, and (4) supported while bodily unsatisfied. The findings highlight the dual nature of successful weight loss, showing both positive and negative consequences that should be considered in intervention planning. Additionally, the qualitative analysis of self-concept contributes to the growing literature in this field. A predominantly positive spontaneous self-concept was observed, suggesting that both dimensions may enhance success, making their understanding crucial for effective interventions.

## Introduction

Obesity, one of the most severe public health concerns in 21st century Europe, has witnessed a rapid surge in prevalence and obesity-related disease burdens (GBD 2019 Risk Factors Collaborators, 2020). Globally, obesity rates nearly tripled between 1975 and 2016, as reported by the World Health Organization (2025). If these prevalence trends persist, it is projected that by 2030, nearly half of the world’s adult population will grapple with overweight or obesity. Individuals afflicted by obesity often contend with a range of medical conditions, including cardiovascular disease, hypertension, type 2 diabetes, musculoskeletal disorders, cancers, and obstructive sleep apnea, among others (Blüher, 2019; Heymsfield and Wadden, 2017; World Health Organization, 2025). Furthermore, individuals may also present psychological distress (e.g. depression, self-esteem; Fabricatore et al., 2011; Rosenberger et al., 2007) triggered by weight-related stigma and discrimination (Ogden and Clementi, 2010; Sánchez et al., 2023; Steptoe and Frank, 2023; Westbury et al., 2023), despite growing societal acceptance and awareness about obesity (Lancsar et al., 2022; Sikorski et al., 2011). This distress is frequently associated with heightened dissatisfaction with one’s body, increased distress levels, a more pronounced fixation on appearance, and a negative body image (Alimoradi et al., 2020; Annis et al., 2004; Latner and Wilson, 2011; Rosenberger et al., 2007). Research has also demonstrated that a greater body weight contributes to higher psychological distress (Steptoe and Frank, 2023).

The investigation has devoted significant efforts developing structured interventions to promote successful weight loss (e.g. Hartmann-Boyce et al., 2022; Johns et al., 2014; Myers-Ingram et al., 2023). Recognized as an essential approach for improving obesity-related complications (Tahrani and Morton, 2022), the definition of a “successful” weight loss underscores the intentional reduction in body weight and the adoption of health-related behavioral modifications (Olateju et al., 2021). It is established that weight loss can be a challenge, and sustaining the achieved results can be even more difficult to maintain (Hall and Kahan, 2018). Maintaining weight loss is crucial in effectively preventing obesity (Karfopoulou et al., 2013). Successful long-term weight loss has been characterized by a decrease in weight of at least 10% of the initial body weight (Wing and Phelan, 2005) maintained for at least 1 year (Institute of Medicine, 1995). However, definitions are unclear, and some authors argue that 5% of intentional weight loss maintained for at least 1 year can be considered a moderated clinically significant weight loss (Montesi et al., 2016). However, weight regain after a successful weight loss remains a prevailing concern (Machado et al., 2022). Some studies have tried to map the strategies with individuals who achieved weight loss maintenance (Montesi et al., 2016; Phelan et al., 2022), although the combination of diet and exercise results in more weight loss over a long-term period (Jakicic et al., 2008; Karfopoulou et al., 2013; Swift et al., 2018).

Several studies show that even modest weight loss can improve body dissatisfaction and body image (Bouzas et al., 2019; Hao et al., 2023; Withnell and Bodell, 2023). In a longitudinal study examining the covariation between satisfaction and weight-loss related outcomes, Baldwin et al. (2009) found that factors beyond mere weight change (i.e. levels of self-control, positive feedback and improvement in clothes fit) are strongly associated with individual’s satisfaction, independent of the actual amount of weight lost.

However, successful weight loss is not exclusively linked to positive consequences (Phelan et al., 2022). Aamodt (2016) reports that most individuals who lose weight experience obsessive thoughts, binge eating, depression, and anxiety, potentially compromising weight maintenance. The author also contends that striving for the desired body image is often an exhaustive, psychologically harmful and counterproductive process. Similarly, Gorin et al. (2007) found that weight-loss-related changes often fall short of expectations, even in individuals who have lost an average loss of 19% of body weight over 2 years. Unwanted increased attention from others (Annis et al., 2004) or undesirable body changes (Sarwer et al., 2011) represent negative consequences. The subject of perceived—primarily negative—consequences of successful weight loss has received limited empirical attention (Alegría and Larsen, 2015; Rosas et al., 2019; Sarwer et al., 2011). Further in-depth research (such as qualitative research) is needed to characterize the experiences of individuals who have achieved weight loss (Santos et al., 2010; Wing and Phelan, 2005), as specific themes and issues may have been overlooked or neglected (Creswell, 2013). In addition, significant research work is primarily or entirely focused on female participants (e.g. Alegría and Larsen, 2015; Draper et al., 2016; Karfopoulou et al., 2013; McMahon et al., 2016).

Beyond perceiving the process of weight change and its consequences, individuals’ self-concept also provides pertinent information about the success of maintaining (or not) weight loss. Linville (1985) defines the *self* as a cognitive representation that organizes relevant self-knowledge. Harter (2008) suggests that the *self* is shaped by cognitive, developmental and social factors, with cognitive skills and socialization experiences playing a vital role in the content and structural dimensions of the self. Oyserman et al. (2012) view the *self* as both a thinking agent (“I think”) and the subject of contemplation (“about me”). The structure of self-concept is large, dynamic and complex (Baumeister, 2005). The dimensions comprising a person’s self-concept vary in prominence over time. Baumeister (2005) identifies *spontaneous self-concept* as the domain presented in one’s mind at a specific moment. Thus, spontaneous self-concept can change, while self-esteem and other core facets of self-concept remain relatively stable.

Considering the diverse dimensions that a global self-concept can encompass (Markus, 1977), the number of self-schemas reflects the roles one assumes in life (being a father, worker or colleague, e.g.; Rosas et al., 2017). Given the impact of health threats on various life domains, self-concept related to physical health holds significant importance (Rosas et al., 2017). Bosc et al. (2022), in a sample of post-bariatric surgery patients, reported a positive correlation between weight loss after surgery and appearance evaluation (e.g. feelings of physical attractiveness or unattractiveness, meaning that patients reported higher satisfaction with one’s appearance) and overall satisfaction with most of one’s body areas. A scoping review (Conradson et al., 2022) showed that improvements in positive psychological well-being (such as self-acceptance, self-esteem, and optimism) are related to weight loss and successful lifestyle changes with or without weight loss.

Global self-esteem is higher in males than females (Bleidorn et al., 2016), although physical appearance (as body cultural pressures) appears more prominent in females than males (Marčič and Grum, 2011). These authors found that females revealed a better overall self-concept (e.g. are more satisfied in their relationship with others, family, and social context). Binkley et al. (2009) also established that participants who perceived themselves as having a healthy weight reported significantly higher physical self-concept scores compared to those who perceived themselves as having overweight or obesity. However, physical self-concept and other domains comprising self-concept have not been thoroughly explored in the context of weight loss.

Hence, this study aims to explore the perceived positive and negative consequences and the spontaneous self-concept of successful weight loss in a sample of Portuguese men and women using a directed qualitative content analysis approach.

## Materials and methods

### Procedure

This study is part of a larger investigation called WELCOM—Weight Loss in the Community, which focuses on obesity and successful weight loss. The research was conducted at the William James Center for Research, Ispa—Instituto Universitário.

The study’s sample was gathered from clinical (e.g. Hospital de Santa Maria—Centro Hospitalar Universitário Lisboa Norte, EPE) and community settings (e.g. ADEXO—Association of Obese and ex-Obese Patients; social networks). After obtaining approval by the Ethics Board of Hospital de Santa Maria, the researchers presented the aims and procedures of the investigation to healthcare professionals. Subsequently, healthcare professionals/workers from the associations established the initial contact with potential participants and referred those who expressed interest in participating and granted authorization for their contact information to be shared. The researchers then contacted participants recruited through community and clinical settings (either by telephone or email, according to individuals’ preferences) to confirm the inclusion criteria through a triage questionnaire. Afterward, the informed consent, as well as the authorization for the audio recording of the interview, were sent via email, and subsequently, the interview was arranged at a convenient time.

All participants received informed consent and signed written permission for the audio-recorded interview. Interviews conducted in person took place in a private room with a closed door in the clinical setting, ensuring a confidential individual context. When conducted by telephone, the researcher confirmed that the interview occurred in a private room with a closed door, guaranteeing appropriate timing and physical context. Trained researchers interviewed all participants and were supervised by the principal researcher. No significant incidents occurred during the interviews. The collected data was meticulously transcribed, encompassing audible and non-verbal elements, using MAXQDA software (version 22.7.0). This research adhered to the guidelines the Order of Portuguese Psychologists (2011).

### Participants

The sample comprised 60 individuals (22 men and 38 women), with an average age of 41.96 years (SD = 13.06 years) and with an average total weight loss of 24.40% (SD = 12.44%). Body Mass Index (BMI) data a mean BMI of 25.58 kg/m^2^ (SD = 2.32 kg/m^2^). The most prevalent weight-loss method (Table 1) was changes in eating habits (35.2%), followed by changes in eating habits combined with physical exercise (33.3%) and bariatric surgery (11.1%).

**Table 1.** Frequencies and percentages of socio-demographic and health-related characteristics.

Socio-demographic and health-related characteristics	Frequency	Valid %
Biological sex		
Men	22	36.7
Women	38	63.3
Relationship status		
With an affective-sexual relationship	40	70.2
Without an affective-sexual relationship	17	29.8
Missing	3	—
Parity		
Without children	16	31.4
1 or 2 children	27	52.9
3 or more children	8	15.7
Missing	9	—
Educational level		
Primary school	4	7.5
Middle school	7	13.2
High school	9	17.0
College degree	33	62.3
Missing	7	—
Professional status		
Active	42	82.4
Inactive	9	17.6
Missing	9	—
Annual household income		
10,000€ or less	11	22
10,001€–20,000€	10	20
20,001€–37,500€	13	26
37,501€–70,000€	9	18
More than 70,001€	7	14
Missing	10	—
Physical exercise practice		
No	12	21.4
Yes	44	78.6
Missing	4	—
Recent disease		
No	41	73.2
Yes	15	26.8
Missing	4	—
Recent psychological problem		
No	46	82.1
Yes	10	17.9
Missing	4	—
Used weight-loss methods		
Bariatric surgery	6	11.1
Changes in eating habits	19	35.2
Physical exercise	2	3.7
Bariatric surgery and changes in eating habits	1	1.9
Changes in eating habits and physical exercise	18	33.3
Bariatric surgery, changes in eating habits and physical exercise	4	7.4
Changes in eating habits and other	3	5.6
Other	1	1.9
Missing	6	—

Inclusion criteria encompassed: (a) being at least 18 years or older; (b) possessing minimum literacy skills and being able to participate in an extensive audio-recorded interview; (c) having a weight loss equal to or above 7% of the initial body weight; (d) sustaining the weight loss for a minimum of 12 months; and (e) having a BMI below 30 kg/m^2^.

### Measures and materials

A questionnaire was administered to participants to evaluate socio-demographic characteristics (e.g. age, sex, educational level), physical exercise (e.g. *Do you practice any physical activity? If so, how many times per week?*), physical (e.g. *Have you recently had any disease?*), and psychological health (e.g. *Have you recently had any psychological problem?*).

Semi-structured interviews, developed by the research team, were conducted to address pre-defined deductive categories—spontaneous self-concept and perceived positive and negative consequences of successful weight loss—and encompassed the questions posed to the participants. The interview protocol comprised the following questions and respective prompts:

1. Negative Perceived Consequences
  - Did significant weight loss and maintaining this lower weight have any negative consequences? If so, what were they?
  - Did you experience any negative consequence on a social level?
  - What about on a professional level? If so, could you elaborate further?
  - And on a family level? Do you have children? If so, do you think your current weight has impacted how you relate or interact with them?
  - What about on a personal level (such as your emotional well-being)? And on a sexual or intimate level (such as in your relationship with a partner or your confidence in intimate situations)? If so, could you explain further?
2. Positive Perceived Consequences
  a. Do you think that weight loss and maintaining this new lower weight had any positive consequences? If so, what were they?
  b. Did it have any positive consequences on a social level (in terms of how you plan your activities and interact with your friends)? If so, could you tell me a bit more about that?
  c. What about on a professional level? If so, could you elaborate a bit more?
  d. And on a family level (in terms of how you interact with your family)? Do you have children? If so, do you think your current weight, lower than your previous weight, influences how you interact and relate to them?
  e. What about on a personal level? Do you feel your weight affects your personal life? And on a sexual level?
3. Spontaneous Self-Concept
  a. How do you think acquaintances (people you know but are not close to) currently perceive you?
  b. And what about those closer to you?
  c. How does your family perceive you?
  d. How do your friends perceive you?
  e. And how do you see yourself? How would you describe yourself? How do you evaluate yourself in relation to what defines you?

### Data analysis

The data collected from the semi-structured interviews was transcribed in Portuguese and analyzed using a deductive and directed qualitative content analysis. The constructs explored—Negative and Positive Consequences, and Spontaneous Self-concept—were derived from established literature and theories, including the Common-Sense Model of Self-Regulation (Leventhal, Phillips and Burns, 2016) and the Health Belief Model (Champion and Skinner, 2008; Rosenstock, 1974). These models emphasize the significance of constructs like consequences (or benefits and barriers) in understanding behavior change. Furthermore, authors such as Baumeister (2005), highlighted the role of spontaneous self-concept as a potential factor that can either facilitate or hinder behavior change.

As outlined by Hsieh and Shannon (2005), the directed content analysis technique follows a theory-driven deductive approach. This method facilitates the identification of relevant interview segments and the categorization of data based on predefined themes. These initial themes, derived from prior research, theoretical frameworks, or the study’s objectives, were iteratively refined during the analysis to enhance their relevance and accuracy.

The study’s macro-categories, corresponding to its core areas of inquiry, were predefined based on an extensive literature review. While these categories—(1) negative perceived consequences, (2) positive perceived consequences, and (3) self-concept—formed the analytical foundation, subcategories emerged through a systematic and iterative process. The development of subcategories involved: (a) establishing pre-existing categories aligned with the study’s core themes; (b) creating and defining emergent subcategories as justified by the content’s manifest nature. These subcategories were derived from the pre-existent categories; (c) generating personalized codes to systematically classify the data within each category; (d) identifying and coding specific speech segments to ensure consistency, coherence and depth in the analysis.

To ensure methodological rigor, two investigators (CD and RR) initially developed an analytic grid. This grid underwent further refinement through iterative discussions with two additional investigators (IQG and FP). The collaborative coding process included regular cross-checking to ensure the subcategories moved beyond surface-level patterns and accurately represented content emerging from participants’ responses. Subcategories were coded deductively, maintaining a balance interpretative depth and adherence to the data.

Reliability was a crucial measure for ensuring confidence and accuracy in the findings. Two researchers independently coded the same pair of transcribed interviews (the coding of two interviews) using the developed codification grid. Cohen’s kappa coefficient, calculated at 0.734, indicated substantial agreement, validating the consistency of the analytical process. This rigorous and collaborative approach ensured that, while macro-categories were predefined, the subcategories emerged as nuanced interpretations deeply rooted in the participants’ experiences.

The representation of subcategories was achieved through frequency and percentage analysis. To uncover systematic relationships and groupings among the identified categories, a Multiple Correspondence Analysis (MCA) was conducted. MCA, often used in exploratory analyses of extensive qualitative study (Costa et al., 2013), enabled the development of an explanatory model of the constructs under investigation.

Consequently, MCA was performed to identify systematic relationships between categories derived from the data, producing an explanatory model of the inherent constructs (Beh, 2004). Descriptive analysis and the explanatory factor model were developed using the software IBM SPSS Statistics (v. 29, IBM Corp., Armonk, NY). These analyses provide critical insights into the interrelationships among categories offering a comprehensive understanding of the constructs explored in this study.

In addition to the qualitative analysis, an exploratory quantitative analysis was conducted to examine potential differences in the frequency of perceived consequences (positive and negative) between two groups: participants who lost more than the average weight loss of the sample (≥24.4%) and those who lost less (<24.4%). Frequencies and percentages were calculated for each consequence domain within both groups.

## Results

### Perceived consequences

The first deductively determined category—*Negative Perceived Consequences* of the successful weight loss—encompassed consequences from multiple domains that the participants expressed as having a negative impact on themselves due to the achieved weight loss. The emergent negative consequences categories (presented in Table 2) included references to the following:

1. *Family*, which entailed the following sub-categories: exacerbation of pre-existing relational problems/emergent relational problems; ill-tempered/irritability perceived by others; jealousy from partner; incomprehension from family regarding the change in eating behavior patterns; and worry with excess thinness.
2. *Body Image*, which states the undesirable effects on one’s physical image and includes the sub-categories of: flaccid skin; ill-looking appearance; decreased breast size; and loss of muscular mass.
3. *Psychological*, which included the following sub-categories: perceived depreciation (of others) toward the successful weight loss; frustration due to food restriction; obsession with weight loss; confrontation with failed expectations; fear of not being loved; perception that the successful weight loss caused strangeness among others; restriction of alcohol consumption; and deceleration in the weight loss process despite the same effort.

**Table 2.** Frequencies and percentages of the perceived consequences of a successful weight loss.

Categories/Sub-categories	Example	*n*^a^ (NM)^b^	*n*%^c^
*Negative consequences*			
Family	“Yes, the jealous husband”	10 (22)	16.7
Body image	“Even though I also have some loose skin now…”	9 (12)	15
Psychological	“It was a different kind of look. They didn’t seem to appreciate what I was achieving”	12 (42)	20
*Positive consequences*			
Family			
Weight loss satisfaction	“My own family, to me, is more satisfied to see me like this, because I was obese before, and now I have managed to return to my previous state, and I have been able to maintain it over the years”	7 (13)	11.7
Body image			
Body image improvement	“(…) it involved a bit more of looking in the mirror and liking what we see.”	17 (21)	28.4
Pleasure in appearance-care	“(…) I feel that the clothes fit me better.”	12 (20)	20
Easier clothing acquisition	“(…) there were also some clothes that I no longer wore and I started wearing them again.”	11 (21)	18.3
Volume decrease	“(…) now, as I have greatly reduced my circumference, I feel good.”	6 (11)	10
Psychological			
Increased self-esteem	“I gained a lot of self-esteem.”	30 (52)	50
Increase subjective well-being	“(…) I feel so much better.”	20 (25)	33.3
Increased self-confidence	“(…) and it changed my confidence a lot, very, very much, I am aware of that (…)”	10 (20)	16.7
Identity-role model	“My aunt started following the same meal plan that I had followed.”	7 (25)	11.7
Increased self-efficacy	“(…) If I want to travel, I will travel.”	6 (9)	10
Health			
Mobility/agility improvement	“A person starts to feel more agile, healthier because when you’re heavy, you can’t move.”	13 (30)	21.7
General health gains	“I gained health.”	11 (15)	18.3
Sexual			
Sexual disinhibition	“It’s not so hard for me to be undressed with the lights on (…)”	15 (20)	25
Sexual without specification	“For the better, much better.”	6 (6)	10
Social			
Positive comments and reactions	“I receive more compliments than I used to.”	27 (68)	45
Sociable	“I am a more sociable person now.”	12 (18)	20
Informal and emotional social support	“My mother gave me a lot of strength.”	11 (16)	18.3
*Negative spontaneous self-concept*			
Body image	“I always see myself as a chubby and fat person.”	17 (63)	28.3
*Positive spontaneous self-concept*			
Body image	“I have a better image of myself.”	50 (175)	83.3
Global self-esteem	“I feel, at this moment, I can tell you that I feel very good about myself.”	16 (26)	26.7
Explicit personality	“I see myself as a more confident person.”	40 (157)	66.7
Interpersonal	“I continue to be as sociable as I was.”	6 (11)	10

a Participants who mentioned the category/sub-category, considering the total sample (*n* = 60).

b Total number of times the sub-category was mentioned.

c Percentage of participants who mentioned the category/sub-category.

The second deductively identified construct—*Positive Perceived Consequences* of successful weight loss—also comprehended diverse categories featuring the positive impacts following weight loss. In this category, emergent consequences were categorized into the following domains:

1. *Family*, which covered the following sub-categories: family satisfaction with the accomplishment of weight loss; capacity/willingness to accompany family members in activities that were not previously participated in; processes related to successful weight loss (e.g. nutrition, exercise, better time management) that led to family bonding/closeness; identification of general gains; and demystification about weight loss.
2. *Physical*, which entailed the following sub-categories: satisfaction with an improved body image; pleasure in appearance-care; greater ease in acquiring clothing; volume reduction; positive report of being unrecognizable, especially in the eyes of others; perception (self or by others) of looking younger; achieving good results after surgery; and feeling physically fit after successful weight loss.
3. *Psychological*, which comprised the following sub-categories: increased self-esteem; increased subjective well-being; increased self-confidence; perceiving oneself as a role model (e.g. to friends, family); perception of generalized self-efficacy; improved mood; increased self-control; cessation of discrimination; restructuring of useless cognitions; pleasure in activities unrelated to body and weight; general gains; and satisfaction for having achieved the goal.
4. *Health Behaviors*, which encompassed the following sub-categories: improved mobility, locomotion, and agility; identification of general health gains, but without specifying which ones; reduction or disappearance of pain; positive sensations or experiences at the physical/body level; increase in overall quality of life; increase in metabolism speed; reduction of cholesterol levels; improvement in the digestion process; enhancement in sleep quality; and better skin.
5. *Couple relationship*, concerning quality improvement in the couple relationship, and positive sexual consequences, which included the following sub-categories: disinhibition in intimate/sexual interaction, general sexual gains; overall increase in sexual satisfaction; and increase in sexual desire.
6. *Social*, which entailed the following sub-categories: positive comments and reactions, including constructive feedback, affirmation, and social comparison; higher pleasure in socializing and enjoying the company of others; informal and emotional support from others (e.g. empathy, trust, and affection); formal and informative support (e.g. advising on maintaining the lost weight); pleasure in going out; formal assistive and instrumental social support with goods and services that directly assist the person; satisfaction in triggering envy among friends due to their body image; capability of performing tasks (perceived by others); and valuing pre-existing interpersonal relationships.

### Spontaneous self-concept

Multiple categories emerged when it was asked to the individual about how he/she thinks others—acquainted without a close relation, family and friends—see him/her and how the person sees oneself (i.e. construct of spontaneous self-concept).

The first deductively category—*Negative Spontaneous Self-Concept*—involves how one sees oneself from a bodily perspective. The emergent negative category (Table 2) was categorized into the following domain:

1. *Body Image*, concerning undesirable features which covered the following sub-categories: body image dissatisfaction; disregard/disinterest in the successful weight loss by others; evaluation of the body as excessively thin; dissatisfaction regarding the belly/abdomen/stomach; having an overweight image; appearance of illness; negative evaluation of the body associated with the aging process; wrinkles increase perception after the successful weight loss; and dissatisfaction with the body shape.

The second deductively generated category—*Positive Spontaneous Self-Concept*—refers to a person’s beliefs, thoughts and feelings about their identity, worth, and abilities in a favorably manner. The emergent positive category was categorized into the following domains:

1. Body image, which included the following sub-categories: body image satisfaction; pleasure in appearance; perceiving oneself as thinner after successful weight loss; body investment; perception of weight loss; perceived by others as unrecognizable; more beautiful; youthful; feeling more fit; more elegant; acceptance of the body; skillful; and attractive after successful weight loss.
2. *Global self-esteem*, signifies the affective and evaluative dimension of the self-concept without a specific ambit.
3. *Explicit personality*, representing conscious awareness which entailed the following sub-categories: valued; persistent; confident; happy; optimistic; good-humored; thoughtful; organized/methodical; courageous; extroverted; perfectionist; energetic; calm; grateful; intelligent; self-efficacious; trustworthy; flexible; reserved; and responsible.
4. *Interpersonal*, entailing both sympathetic (perceiving oneself as someone who provides support and helps others) and sociable (perceiving oneself as sociable, flexible, and adaptable in social contexts) sub-categories.

The frequencies of the categories/sub-categories (derived from both spontaneous self-concept, and negative and positive perceived consequences of a successful weight loss) are detailed in Table 2. In adherence to criteria, 25 emergent (sub)categories were mentioned by a minimum of six participants (representing 10% of the sample) were considered.

The explanatory model was initially explored with a six-factor and, afterward, a five-factor structure; which were meticulously analyzed. Ultimately, a four-factor model provided a satisfactory solution, aligning with both the existing literature on perceived consequences and spontaneous self-concept in the context of weight loss process and the analysis performed (namely, higher factor loadings of each category in their respective factors). The results of the MCA for the perceived consequences and spontaneous self-concept of successful weight loss are displayed in Table 3.

**Table 3.** Four-dimensional model of the perceived consequences and spontaneous self-concept of a successful weight loss.

Categories/sub-categories	All gains (health, psychological and social) but the body image (α = 0.67)	Role-model and bodily efficacious with fallouts (psychological and family) (α = 0.65)	Body satisfaction and sexual disinhibition (α = 0.55)	Supported while bodily unsatisfied (α = 0.48)
*Negative consequences*				
Family	0.10	0.41	0.10	0.04
Body image	0.00	0.04	0.00	0.24
Psychological	0.00	0.36	0.14	0.00
*Positive consequences*				
Family				
Weight loss satisfaction	0.26	0.01	0.02	0.28
Body image				
Body image improvement	0.00	0.00	0.07	0.01
Pleasure in appearance-care	0.02	0.10	0.53	0.04
Easier clothing acquisition	0.08	0.23	0.00	0.19
Volume decrease	0.00	0.33	0.23	0.05
Psychological				
Increased self-esteem	0.20	0.00	0.06	0.00
Increased subjective well-being	0.15	0.03	0.00	0.00
Increased self-confidence	0.25	0.02	0.03	0.00
Identity-role model	0.02	0.09	0.06	0.00
Increased self-efficacy	0.06	0.41	0.08	0.00
Health				
Mobility/agility improvement	0.14	0.00	0.06	0.14
General health gains	0.24	0.01	0.01	0.03
Sexual				
Sexual disinhibition	0.01	0.03	0.04	0.00
Sexual without specification	0.00	0.01	0.16	0.16
Social				
Positive comments and reactions	0.06	0.03	0.01	0.01
Sociable	0.21	0.16	0.09	0.03
Informal and emotional social support	0.02	0.20	0.13	0.36
Negative spontaneous self-concept				
Body image	0.42	0.04	0.08	0.07
*Positive spontaneous self-concept*				
Body image	0.02	0.05	0.01	0.00
Global self-esteem	0.24	0.01	0.09	0.00
Explicit personality	0.24	0.00	0.10	0.16
Interpersonal	0.06	0.09	0.02	0.05
Eigenvalue	2.81	2.66	2.12	1.86
Variance explained by factors (%)	11.23	10.62	8.49	7.43

There were group differences in the frequencies of specific consequences reported. Table 4 presents the distribution of positive and negative consequences by weight loss group, highlighting differences in areas such as family dynamics, body image, psychological effects, and social reactions. Participants were categorized into two groups based on their percentage of total weight loss: a lower weight loss group (<24.4%, *n* = 31), and a higher weight loss group (≥24.4%, *n* = 25).

**Table 4.** Frequency of reported positive and negative consequences by weight loss groups.

Categories/Sub-categories	<24.4% Group	≥24.4% Group
*Negative consequence*	*N* (Valid %)	*N* (Valid %)
Family	2 (6.5%)	8 (32%)
Psychological	4 (12.9%)	7 (28%)
Body image	2 (6.5%)	6 (24%)
*Positive consequence*	*N* (Valid %)	*N* (Valid %)
Family		
Weight loss satisfaction	3 (9.7%)	4 (16%)
Body image		
Body image improvement	10 (32.3%)	6 (24%)
Pleasure in appearance-care	6 (19.4%)	5 (20%)
Easier clothing acquisition	6 (19.4%)	3 (12%)
Volume decrease	1 (3.2%)	4 (16%)
Psychological		
Increased self-esteem	17 (54.8%)	11 (44%)
Increase subjective well-being	12 (38.7%)	6 (24%)
Increased self-confidence	5 (16.1%)	4 (16%)
Identity-Role model	4 (12.9%)	3 (12%)
Increased self-efficacy	3 (9.7%)	2 (8%)
Health		
Mobility/agility improvement	4 (12.9%)	9 (36%)
General health gains	6 (19.4%)	5 (20%)
Sexual		
Sexual disinhibition	9 (29%)	5 (20%)
Sexual without specification	1 (3.2%)	4 (16%)
Social		
Positive comments and reactions	17 (54.8%)	9 (36%)
Sociable	4 (12.9%)	7 (28%)
Informal and emotional social support	5 (16.1%)	6 (24%)

## Discussion

The majority of individuals who lose weight begin to regain the lost kilograms within approximately 1 year (Neufeld et al., 2012). Consequently, alongside weight loss, the treatment of obesity prioritizes long-term maintenance, making it essential to comprehend the contributing factors to successful weight loss.

In line with this, the present study sought to comprehensively identify and explain the perceived consequences (both positive and negative) and the characteristics of spontaneous self-concept (how one perceives oneself and how he/she thinks others perceive him/herself) in 60 individuals who achieved successful weight loss.

The emergence of positive consequences and their diversity proved notably greater than negative consequences. This suggests that the weight loss process often brings about more positive experiences than negative ones. These findings are consistent with the study by Wing and Hill (2001), which suggested that weight loss maintenance tends to enhance most individuals’ overall quality of life. Given that the participants in this study had achieved successful weight loss and were in a maintenance phase, the higher number and impact of positive consequences could significantly promote successful maintenance. Conversely, a study by Jackson et al. (2014) proposed that the personal costs of weight loss may outweigh the benefits. The magnitude of the personal cost may be computed not only by the number of losses but also by the relative importance of each loss. Although the present study did not assess the relative impact of each consequence, it shows that: (1) negative consequences are in less number than positive and (2) this is observed in a sample of adults who have been able to maintain the weight loss along an extended period (12 months). Thus, positive consequences are expected to be a compiling force in promoting successful maintenance.

Within the positive perceived consequences, the most prevalent category in the family domain was *weight loss satisfaction* (11.7%). Regarding the body image domain, the sub-category related to *body image improvement* (28.4%) was the most mentioned. Literature indicates that even modest weight loss leads to body dissatisfaction and body image improvement (Foster et al., 1997). Similarly, long-term studies (12 or 24 months) have shown that there is a body image improvement after bariatric surgery, with studies reporting a higher satisfaction rate within 48 months after bariatric surgery than did in pre-operatory (Sarwer et al., 2015, 2018; Zeller et al., 2011).

Additionally, the most prevalent positive perceived consequences were found in the psychological domain: *increased self-esteem*, mentioned by 50% of the sample, is consistent with other studies (e.g. Kinzl et al., 2003; Wadden et al., 1996); and *increased subjective well-being* (33.3%), was also found in line with previous studies (e.g. Swencionis et al., 2013; Wing and Hill, 2001).

In the health sphere, only positive consequences were reported, suggesting that successful weight loss generally has an optimistic impact on physical health. Participants perceived a *mobility/agility improvement* (21.7%) and *general health gains* (18.3%). Alegría and Larsen (2015) found similar health and energy level improvements among individuals who successfully lost weight. Additionally, enhanced physical well-being, reduced pain, and improved quality of life were also mentioned. Wing and Hill (2001) reported that over 90% of their sample experienced improved quality of life, energy levels, and mobility, with 85.5% showing enhanced physical health. Mobility increased by around 70% alike emerged in a study by Crisp et al. (1977).

Regarding the sexual sphere, again, only positive consequences were identified, with an emphasis on *disinhibition*. This sub-category suggests that weight loss and improved body image lead to a more positive self-perception, increased comfort, and confidence during sexual activities, both with a partner and individually. Existing literature indicates that higher levels of well-being are linked to better functioning and sexual satisfaction (e.g. Ackard et al., 2000; Impett and Tolman, 2006). Another contributing factor is underlined by Gilmartin et al. (2015): an improved body image positively influenced participants’ sexual well-being, making them more active and dynamic in intimate relationships and attracting sexual attention.

In the social domain, the most mentioned sub-category was *positive comments and reactions* (45%). Regarding social consequences, a qualitative study by Hindle and Carpenter (2011) emphasized the significance of social support in successful weight loss, supported by Anderson et al. (2007). Baldwin et al. (2009) presented a longitudinal study indicating that many weight-loss-related consequences, such as perceived attractiveness and positive feedback (e.g. positive comments), contributed to people’s satisfaction. Anderson et al. (2007) also highlighted the link between social support and self-efficacy, which in turn arises from a sense of self-esteem (Cochrane, 2008), an emergent content also evident in this study (positive consequence—mentioned by 50%). Moreover, on the social consequences, it is noteworthy that successful weight loss led several participants to become more *sociable* (20%). A study by Alegría and Larsen (2015) found that participants reported increased confidence and ease in social interactions.

Despite the positive consequences, the perceived negative consequences identified in this study constitute an important result and should not be overlooked. Successful and desired weight change also has drawback/costs and these are significant as they might be associated with the frequently reported outcome of weight regain. As emphasized in previous studies, interventions should focus not only on the perceived benefits or gains (which might contribute to the motivation and meaning attribution of the behavior change), but attention must also be paid to the associated costs or losses, as they might contribute to relapse into previous eating/activity patterns (Hartmann-Boyce et al., 2023; Madigan et al., 2015). Considering categories reported by at least 10% of the sample, family, body image, and psychological domains were mentioned within the context of negative weight loss outcomes. As for negative family consequences, mainly marital problems arise from jealousy and relational issues. These findings are supported by the literature, which states that people who mention negative repercussions refer to the increase in marital discord and jealousy as negative effects. A possible interpretation is that for some husbands, it was not pleasing that their wives became more attractive and active, especially when they began to spend less time at home due to their social commitments (Kinzl et al., 2003). In small numbers but still present, some of the negative psychological consequences identified in this study are related to the perception of devaluation by others. And if on one hand compliments from family members, friends, or even acquaintances can be a source of motivation for the weight loss process (Baldwin et al., 2009), the perception of friends or family, in this case experienced by the individual as indifference or devaluation, can constitute possible obstacles to achieving/maintaining this goal (Hammarström et al., 2014). On the other hand, in a weight loss context, especially when significant, it is common for patients to experience body dissatisfaction due to excess skin on the abdomen, arms, face, and thighs (Kinzl et al., 2003; Swan-Kremeier et al., 2005), a factor that is consistent across any method of weight loss. However, it is often those who have lost weight through surgery who feel the most dissatisfied (Alegría and Larsen, 2015). The most likely cause for this point of dissatisfaction is related to the fact this excess skin can cause discomfort and difficulties related to appearance (Kinzl et al., 2003).

When analyzing perceived consequences by weight loss magnitude, distinct patterns emerged. Participants in the higher weight loss group were consistently more likely to report negative consequences, particularly within the family (32% vs 6.5%), psychological (28% vs 12.9%), and body image (24% vs 6.5%) domains. This indicates that greater weight loss may introduce or intensify relational, emotional, or physical appearance-related challenges. Conversely, positive consequences were prevalent across both groups. Notably, health-related gains such as improved mobility were more frequently reported in the higher-loss group (36% vs 12.9%), while psychological benefits (e.g. increased self-esteem, well-being) and positive social feedback were common in both groups, though slightly more pronounced in the lower-loss group (e.g. self-esteem: 54.8% vs 44%). These findings suggest that while greater weight loss may amplify certain benefits, it may also heighten vulnerabilities, particularly in relational and psychological domains, highlighting the need for tailored support in the aftermath of substantial weight loss. This duality underscores the importance of not only promoting weight loss but also addressing its complex emotional and social repercussions.

While spontaneous self-concept tends to be relatively stable over time (Baumeister, 2005), experiences like successful weight loss can introduce fluctuations and changes in the self-representation. Thus, it is reasonable to observe that the categories identified in the consequences align with those reported in the domains of spontaneous self-concept, even if with differing percentages. *Global self-esteem* (26.7%) was perceived as a positive aspect of spontaneous self-concept, reflecting not only how one sees oneself but also how they feel about these self-perceptions, specifically self-esteem—an evaluative dimension of self-conception (Baumeister, 2005).

Another dimension of the self-concept which is frequently impacted with weight change, that is, body image, expresses the degree of cognitive, emotional, and behavioral importance attributed to self-assessment of one’s body (Cash, 2011; Cash and Pruzinsky, 2004). The positive *Body image* (83.3%) references outnumbered negative *Body image* (28.3%), in the spontaneous self-concept. This bolsters the hypothesis that prolonged consequences become internalized, becoming integral elements of spontaneous self-concept. However, it’s important to acknowledge the potential reverse relationship (i.e. participants with positive body image could more easily recognize positive consequences associated with appearance). Nonetheless, after a successful weight loss, negative body image lingers in about a quarter of the sample. It’s possible association with unmet outcome expectations and subsequent weight regain might be important to explore in future studies, in order to more effectively promote healthy weight maintenance.

Weight losses could lead to gains not only in weight and body image but also in other dimensions. For instance, participants described positive assessment toward personality traits, meeting the construct of *explicit personality* (66.7%; i.e. refers to the traits and characteristics that an individual consciously recognizes and can describe about themselves, are deliberately formed, often through self-report measures; Schmukle and Egloff, 2005) and *interpersonal* relationship with others (10%).

The four-dimensional model of the perceived consequences (both positive and negative) and spontaneous self-concept shed light on the profiles of this sample’s successful weight losers. The first factor—“All Gains (Health, Psychological and Social) but the Body image”—indicated an association between those mentioned above, that is, participants who perceived positive consequences associated with health (e.g. general health gains), psychological (e.g. increased self-esteem) and social (e.g. sociable) domains, were those who still had a negative body image. The second factor—“Role-model and Bodily Efficacious with fallouts (psychological and family)”—incorporated both negative (family and psychological) and positive features. This factor shows how participants who perceived a positive body shape (namely, easier clothing acquisition and volume decrease) were also those who recognized themselves has having gained self-efficacy subsequent to the weight loss process, and represent themselves as a role model in weight loss and social/sympathetic (interpersonal sub-category). Thus, appears to be an association between a satisfaction with body shape (despite the negative family and psychological consequences) and turning to others (possibility as a source of compensatory support). The third factor—“Body Satisfaction and Sexual Disinhibition”—demonstrated an association between two pleasurable experiences: pleasure in appearance-care and sexual disinhibition (both positive consequences). Finally, the fourth factor—“Supported while Bodily Unsatisfied”—combined negative body image consequences with family-related positive consequences (weight loss satisfaction), sexual (without specification), and social (informal and emotional social support) consequences.

This study aimed to comprehend perceived consequences, particularly positive factors contributing to weight loss maintenance, as well as potential barriers to weight loss maintenance presented by negative consequences. Furthermore, the study aimed to explore spontaneous self-concept and its association with the perceived consequences, to foster an in-depth understanding of the profile of these successful weight losers.

### Limitations and future directions

While the frequency of mentions attributed by each participant can offer insights into the significance and impact of specific consequences, this approach might oversimplify the overall picture (e.g. a participant might mention numerous positive consequences alongside a single negative one, yet the negative could carry a more substantial impact; in light of this, a clinical context during successful weight loss should delve into the emerging consequences, carefully scrutinizing gains and losses and respective importance, as well as perceived barriers). Consequently, it becomes imperative for future studies to delve deeper into understanding the relative importance and influence of these consequences for the individual change his/her weight. Another limitation is the absence of objective weight assessment and the variability of the interview format (face-to-face or telephone).

It’s worth noting that the existing body of research exploring negative and positive consequences and one’s self-representation (spontaneous self-concept) following successful weight loss needs to be improved, especially using in-depth audio-recorded interviews. In this regard, this study strives for understating the richness and diversity of the identified content. Notably, the prevalence of positive consequences far outweighs the negative ones, potentially signifying that these positive factors play a pivotal role in facilitating successful maintenance. Similarly, how individuals perceive themselves might hold a fundamental role in reinforcing their behaviors. Considering the stark contrast in the number of positive self-concept characteristics compared to negative ones, individuals are more likely to adopt behaviors conducive to weight loss maintenance. Nonetheless, knowing weight regain can happen after 2–3 years after the weight loss (Cooper et al., 2010), future studies might need to adopt a more extended period to grant more robust success criteria.

When these perceived consequences are viewed through a positive lens, they can profoundly influence on the behavioral modifications underpinning the weight loss journey. They can also shape intrapsychic experiences (e.g. attribution of meaning, reshaping beliefs, valuing results, emotional experiences, among others), and such consequences become a driving force for motivation, thereby as a crucial mechanism of action (Palmeira et al., 2023; Teixeira et al., 2015).

The exploration of self-concept holds profound relevance in comprehending how individuals perceive themselves as a whole and in the moment (spontaneous self-concept) following a successful weight loss. Given that perceived bodily changes often carry more significance than actual shifts in body image (Martin Ginis et al., 2012), this aspect can serve as a therapeutic focal point. In clinical practice, addressing potential disparities between the success of weight loss and an individual’s self-perception becomes vital. Future studies should explore the specific impact of each gain/loss following a successful weight loss to understand better which informs maintenance or weight regain.

## Supplemental Material

sj-docx-1-hpq-10.1177_13591053251348301 – Supplemental material for Unveiling the complex landscape of successful weight loss: Perceived consequences and spontaneous self-concept insights Supplemental material, sj-docx-1-hpq-10.1177_13591053251348301 for Unveiling the complex landscape of successful weight loss: Perceived consequences and spontaneous self-concept insights by Filipa Pimenta, Inês Queiroz-Garcia, Cátia Damião, Raquel Rosas and Isabel Leal in Journal of Health Psychology
