# Supplementary material for: Unveiling the complex landscape of successful weight loss: Perceived consequences and spontaneous self-concept insights
Source: J Health Psychol. 2025 Jul 16;31(4):1389–409. doi: 10.1177/13591053251348301 (PMC12960774; doi:10.1177/13591053251348301)
Supplement: sj-docx-1-hpq-10.1177_13591053251348301 – Supplemental material for Unveiling the complex landscape of successful weight loss: Perceived consequences and spontaneous self-concept insights [file sj-docx-1-hpq-10.1177_13591053251348301.docx]

**Supplementary Material**

Original Interview Protocol in Portuguese

1. **Consequências Negativas Percebidas**
2. A perda significativa de peso e a manutenção deste peso mais baixo tiveram alguma consequência negativa? Se sim, quais foram?
3. Teve alguma consequência negativa a nível social?
4. E a nível profissional? Se sim, poderia elaborar um pouco mais?
5. E a nível familiar? Tem filhos? Se sim, acha que o seu peso atual impacta a forma como se relaciona ou interage com eles?
6. E a nível pessoal (como na sua perceção de si mesmo ou bem-estar emocional)? E a nível sexual ou íntimo (como na relação com um parceiro ou na sua confiança em situações íntimas)? Se sim, poderia explicar melhor?
7. **Consequências Positivas Percebidas**
8. Acha que a perda de peso e a manutenção deste novo peso mais baixo tiveram consequências positivas? Se sim, quais foram?
9. Teve consequências positivas a nível social (em termos de como planeia as suas atividades extracurriculares e interage com os seus amigos)? Se sim, poderia falar um pouco mais sobre isso?
10. E a nível profissional? Se sim, poderia elaborar um pouco mais?
11. E a nível familiar (em termos de como interage com a sua família)? Tem filhos? Se sim, acha que o seu peso atual, mais baixo do que anteriormente, influencia a forma como interage e se relaciona com eles?
12. E a nível pessoal? Sente que o seu peso afeta a sua vida pessoal? E a nível sexual?
13. **Autoconceito Espontâneo**
14. Como acha que os conhecidos (pessoas que conhece, mas com quem não tem grande proximidade) o percecionam atualmente?
15. E as pessoas mais próximas de si?
16. Como a sua família o perceciona?
17. Como os seus amigos o percecionam?
18. E como se vê a si próprio? Como se descreveria? Como se avalia em relação ao que o define?
